# Editorial: The effect of musculoskeletal conditions on balance control

**DOI:** 10.3389/fresc.2023.1288322

**Published:** 2023-09-28

**Authors:** Shuaijie Wang, Xinyao Hu, Gonzalo Rivera-Lillo

**Affiliations:** ^1^ Department of Physical Therapy, University of Illinois, Chicago, IL, United States; ^2^ Institute of Human Factor and Ergonomics, College of Mechatronics and Control Engineering, Shenzhen University, Shenzhen, China; ^3^ Physical Therapy Department, University of Chile, Santiago, Chile; ^4^ Neuroscience Department, University of Chile, Santiago, Chile; ^5^ Research and Develop Unit, Los Coihues Clinic, Santiago, Chile

**Keywords:** balance control, fall, muscle strength, physical function, intervention strategies

**Editorial on the Research Topic** The effect of musculoskeletal conditions on balance control

Falls are a global health concern among individuals aged 65 and over (1) exacerbated by age-related declines in balance control, the consequences of falls include musculoskeletal injuries such as fractures, strains, and sprains (2, 3). Hence, investigation of the key factors affecting balance control and fall risk is important due to the increased risk of fall-related injuries. Balance control is a multifaceted process that hinges on the harmonious integration and coordination of diverse sensory, musculoskeletal, and neural systems. Prior investigations have revealed the correlations between factors such as muscle strength, muscle power, joint torque, and joint stiffness with balance impairments and fall occurrences (4–6). These insights have paved the way for the development of interventions.

However, a subset of studies has reported less conclusive outcomes, where physical exercise interventions failed to yield reductions in fall rates or enhancements in balance control. This discrepancy underscores the necessity for a deeper exploration into the influence of musculoskeletal conditions on reactive responses following unexpected perturbations. In light of these complexities and variations, a comprehensive investigation is warranted to reveal the associations between musculoskeletal conditions and balance control and to explore the interventions to improve balance control and lower fall risk.

The research focused on balance control and training in different populations was published in this Research Topic.

Rhynehart et al. investigated the relationship between physical function measures, frailty, metabolic syndrome (MetS), and falls in individuals with complex obesity. The study underscores the connections between frailty, falls, and MetS in individuals with obesity, and reported that fall risk was associated with reduced functional reach and slower sit to stand. While no difference in BMI was found between fallers and non-fallers. The results emphasized the need to enhance physical function in this population for fall prevention.

The study conducted by Bally et al. aims to identify factors associated with falls in both hospitalized and community-dwelling older adults. The research found that falls are influenced by various socio-demographic characteristics and potential risk factors. Malnutrition was found to be associated with an increased risk of falls in older adults, as malnutrition could increase the risk of falling by reduced muscle strength, leading to loss of balance or fall.

Focusing on people with Parkinson’s disease (PD), Mylius et al. investigate the efficacy of dual task training in reducing fall risk. The research compares perceptual and executive dual task training types during high-intensity treadmill training. The study aims to inform therapeutic strategies for balance training in people with PD, emphasizing the importance of tailored interventions to address the intricate challenges associated with fall prevention in this population.

Kamijo et al. examined the effect of postural control exercise in people with stroke, who are at high risk for falls in all poststroke stages. This study explores the impact of upper limb involvement during postural control exercises on the Functional Independence Measure motor items. The research uncovers significant differences in motor items between groups that performed postural control exercises with and without upper limbs. It proposed that postural control practice without the upper limb could improve postural balance in people with stroke in the long-term compared to practices involving the upper limb.

In conclusion, these studies collectively explored the nature of balance control, fall risk, and the necessity of tailored interventions to address these issues across diverse populations. The findings underscore that physical function enhancement, postural control exercise, and personalized intervention could be effective for fall prevention across various contexts.
